# Physiological effects of anthropogenic sound on aquatic animals: where are we and what is next?

**DOI:** 10.1242/jeb.250800

**Published:** 2026-02-11

**Authors:** Ana Širović, M. Clara P. Amorim, Simone Baumann-Pickering, Annebelle C. M. Kok

**Affiliations:** ^1^ Norwegian University of Science and Technology, Trondhjem Biological Station, Bynesveien 46, 7018 Trondheim, Norway; ^2^ MARE – Marine and Environmental Sciences Centre/ARNET - Aquatic Research Network, Departamento de Biologia, Faculdade de Ciências da Universidade de Lisboa, 1149-016 Lisbon, Portugal; ^3^ University of California San Diego, Scripps Institution of Oceanography, La Jolla, CA 92093-0205, USA; ^4^ Leiden University, 2311 EZ Leiden, The Netherlands

**Keywords:** Anthropogenic sound, Sensory perception, Aquatic invertebrates, Fish, Marine mammals

## Abstract

Many aquatic animals have a well-developed sense of hearing as sound is important for communication underwater. However, this trait leaves them susceptible to injury, and physiological and behavioral impacts from exposure to intense or persistent anthropogenic sounds. We provide an overview of the current state of knowledge on the physiological effects of five main sources of anthropogenic sound: marine traffic, seismic exploration, pile driving, other industrial activity and sonar. Our understanding of impacts varies greatly by sound type and taxon, although the studied species do not represent the full taxonomic diversity. Exposure to ship sounds has been best studied in fish and it generally leads to responses along the stress response cascade, while few studies have been conducted on its physiological effect on invertebrates or marine mammals. Effects of exposure to seismic sound show mixed impact across taxa. Pile driving sounds have been shown in captive studies to result in hearing impairment in marine mammals and can cause injury to fishes. Lethal impacts have been documented from naval sonar on marine mammal species. Currently, physiological impacts from other industrial sound sources are poorly documented across taxa. Overall, given the limited number of species examined in sound impact experiments, it is crucial to establish categorizing principles and guidelines and modeled response pathways to improve management strategies, especially as new sound threats continue to emerge in our changing world.

## Teaser

**Summary:** A review of the physiological effects of anthropogenic sound on aquatic animals and a perspective on ways to move this field forward in a strategic way.

## Introduction

Sound is a very important sensory modality for many aquatic animals because it travels through the environment efficiently. Hence, many aquatic animals have a well-developed sense of hearing (Au and Hastings, 2008). Hearing sensitivity and the range of frequencies over which animals can detect sounds vary, but generally, most animals can hear low-frequency (<1 kHz) sounds, and some also have extended hearing capabilities in the ultrasonic range (Au, 1993; Radford and Stanley, 2023; Reichmuth et al., 2013; Richardson et al., 1995; Slabbekoorn et al., 2010; Wartzok and Ketten, 1999; Webb et al., 2008). Odontocetes are the most reliant on and sensitive to such high-frequency sounds, although some pinnipeds and a few fishes are known also to have ultrasonic hearing (Au, 1993; Popper et al., 2004; Reichmuth et al., 2013; Tyack et al., 2011). Reliance on sound makes animals susceptible to changes in their acoustic environment.

Even though the trend is not universal, sound levels from anthropogenic sources in aquatic environments are increasing and their patterns differ from what animals experienced evolutionarily (Andrew et al., 2002; Duarte et al., 2021; McDonald et al., 2006; Miksis-Olds et al., 2018; Širović et al., 2016). Therefore, it is important to consider the possible impact of increasing sound levels, as well as individual high-intensity sounds, on aquatic animals. Many studies on the impact of sound have focused on behavioral responses and masking (e.g. Erbe et al., 2019; Simmons and Narins, 2018; Sivle et al., 2021) or have been limited in taxonomic reach (e.g. Cox et al., 2018; Davies et al., 2024). While these responses often occur in combination, our focus is to summarize the current state of knowledge of the physiological effects of sound exposure across three diverse aquatic groups, invertebrates, fish and marine mammals, along with a discussion of possible future research directions.

Effects stemming from sound exposure depend on the signal characteristics (intensity, frequency components, duration and duty cycle), distance from the source, propagation characteristics and overall length of exposure. Negative physiological effects can include (in order of typically decreasing likelihood as one moves away from the source): death, injury, impacts on hearing ability, primary stress response and additional cascading effects such as metabolic, reproductive or developmental impacts (see Box 4) (Erbe et al., 2018; Southall et al., 2007). Close to the source, multiple effects are likely to occur simultaneously. In many cases, the ultimate response could depend on the animal's behavioral state and health condition (Erbe et al., 2018; Southall et al., 2007).

Impact on hearing is one major type of physiological effect of sound and to understand it better, we first briefly explain the basics of hearing physiology. Specific details of hearing organs vary across taxa, but hair cells are the common auditory sensory receptors that are stimulated by particle motion or acoustic pressure changes, the two components of a sound field (Fay, 1984; Popper and Fay, 1997). In most invertebrates, the sensory organs' statocysts play a role in hearing by detecting particle motion (André et al., 2016). Fish detect sounds with their inner ears, which are also sensitive to particle motion or can have special adaptation to detect sound pressure (see Popper et al., 2021, for a review on fish hearing specializations). Also, the lateral line can have a parallel function for low-frequency particle motion detection (Higgs and Radford, 2013). The basics of hearing physiology in marine mammals are preserved from their terrestrial relatives; they have middle and inner ears and rely on the cochlea for hearing, although their air passages are reduced or filled with fluid, in adaptation to a high-pressure underwater environment (Wartzok and Ketten, 1999). Marine mammals primarily detect and respond to sound pressure. Despite differences in the portion of the sound field that animals detect, impacts on their hearing occur by similar mechanisms across taxa.

The stress response is another common effect of sound exposure and can lead to a cascade of effects. A stress signal is generated by increased production of steroid hormones, including cortisol, corticosterone and aldosterone. Activation of the stress response leads to several behavioral and physiological changes, the latter including secondary stress responses such as increased respiratory rate or metabolic changes (Habib et al., 2001; Sapolsky et al., 2000). Metabolic activity includes all chemical reactions inside cells, but is typically estimated by measuring oxygen consumption or heart rate (Butler et al., 2004). Proxies for oxygen consumption, such as opercular beats in fish or changes in valve closure or locomotory activity in invertebrates can also be used. Many animals suppress their metabolic rate when faced with unfavorable environmental conditions (Richards, 2010; Storey, 1998). While short-term stress responses are adaptive for immediate survival, chronically elevated steroid hormone levels typically have negative fitness consequences (Adamo and Baker, 2011; Dhabhar, 2002; Romero et al., 2009). For example, extended reduction in metabolic rate in species not used to this response can lead to weight loss or increased mortality (Rixon and Stevenson, 1957). Repeated exposure to sound events that result in tertiary stress responses thus has the potential to negatively impact organisms' survival and fitness.

Box 1. Sound measurement terminology Understanding the impact of anthropogenic sounds on marine life requires dealing with complex terminology and units of measurement. Sound pressure levels (SPL) in the ocean are measured by comparing the amplitude of sound pressure to a reference value of 1 µPa and converting to a logarithmic scale with unit decibel relative to 1 µPa (dB re. 1 µPa). The amplitude of sound pressure can be measured in a variety of ways: as the difference between the maximum and minimum peaks over a time period of interest (peak-to-peak, also noted as subscripted pp), or as the difference of zero to maximum peak (zero-to-peak, or 0-p), or by calculating the root-mean-square (rms) of the sound pressure (Madsen, 2005). These measurements result in intrinsic differences in SPL levels; therefore, to enable comparison, it should be noted which measurement was used. We should point out that the reference value used for in-air SPL measurement is 20 µPa. Combined with the difference in acoustic impedance between water and air, this means that SPL values from air and water are not directly comparable.

Noise is a relative term; noise is generally considered to be any signal that is not of interest at a given time. In a sense, noise is any ambient sound, also called ambient noise (Urick, 1983), but in this paper we use the term primarily for sound from anthropogenic sources in the ocean. To measure ambient sound or noise levels, one needs to calculate acoustic energy across a wide band of frequencies. The measurement is typically based on power spectral density, which represents the acoustic power as a function of frequency. This measure requires further normalization by the bandwidth of the signal and thus is typically reported in units of dB re. 1 µPa^2^ Hz^−1^. However, when referring to anthropogenic sound levels, we want to report the source level of the signal in units of dB re. 1 µPa m (Merchant et al., 2015).

Here, we summarize physiological effects from five main sources of anthropogenic sound in the aquatic environment: marine traffic, seismic exploration, pile driving, sonar and other industrial noise. We provide this overview for three main aquatic taxonomic groups: invertebrates, fishes and marine mammals, based on peer-reviewed studies that quantified sound or exposure levels. A summary of these studies, including the sound levels used, is provided in Table S1. We acknowledge that research effort on these questions varies substantially by taxon and across sound sources. As no studies to date have addressed the question of physiological responses to underwater sound in reptiles and amphibians (Simmons and Narins, 2018), we do not discuss those taxa. For mammals, the focus is on marine mammals, including river dolphins. In contrast with past reviews, which have not focused specifically on this topic but instead have often focused on one or two taxa (e.g. Cox et al., 2018; Davies et al., 2024; Erbe et al., 2019; Pieniazek et al., 2023; Popper and Hawkins, 2019; Solé et al., 2023), we highlight similarities and differences across taxa and per sound source, with more details on those sources than typically provided. We hope that this will help stakeholders better understand the broader scope of impacts that a particular source may have across an ecosystem.

## Overview of impacts across sound sources and taxa

**Fig. 1.**
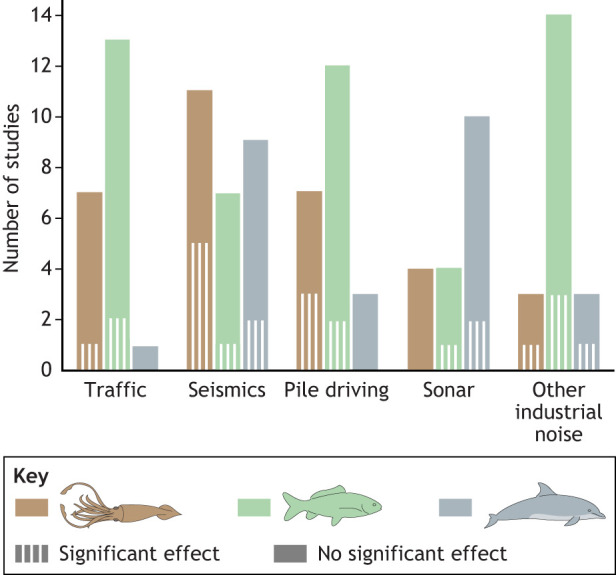
**The number of experimental and observational peer-reviewed studies presented in this Review in which quantitative measures of sound or exposure levels were provided.** Data are shown by sound source and taxon (brown, invertebrates; green, fish; gray, marine mammal studies). Vertical hatching represents studies that reported no significant effect and solid color represents studies reporting a significant effect. Note that in studies with multiple species, if at least one species showed an effect, the study is marked as reporting a significant effect.

**Fig. 2.**
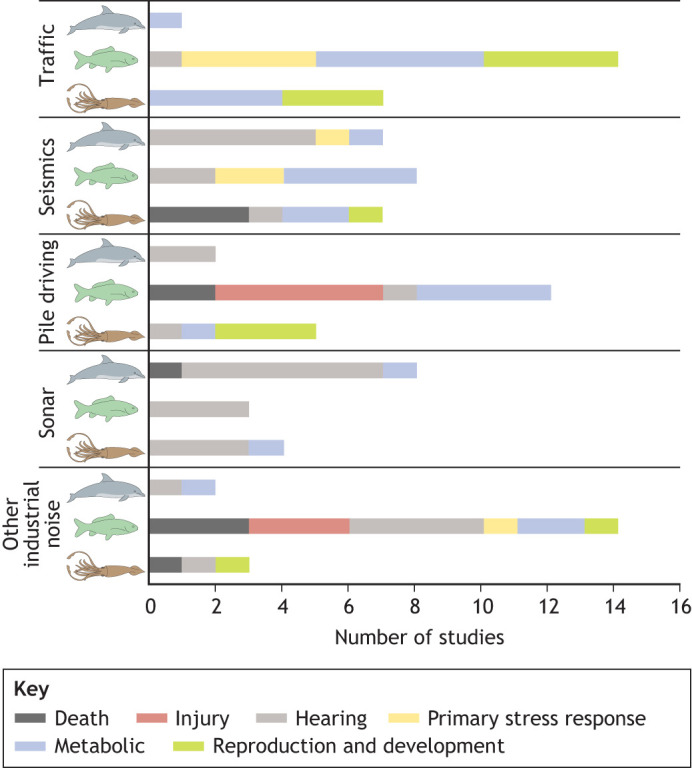
**Number of studies presented in this Review that reported significant results in each category of physiological effect.** Data are shown by sound source and taxon. Effects include: death (black), injury (red), hearing impacts (gray), primary stress response (yellow), metabolic or secondary stress response (blue) and reproductive and developmental or tertiary stress response effects (green). Note that if a study reported more than one type of effect, each is counted separately; hence, the total number of studies per sound type may be larger than the total number of studies with significant effect in Fig. 1.

We narrowed our review of experimental studies to those that included quantitative, consistent measures of sound produced by sources, which is important for understanding the outcomes and resulted in a manageable number of studies per taxon (maximum of 50) or sound source (up to 25). The largest number of such studies has been conducted on fish, reflecting the relative ease of their handling in an experimental environment and long-standing awareness that noise impacts this group (Fig. 1). Generally, studies investigating the impact of impulsive sound (e.g. pile driving, seismics) often documented death or injury as well as impacts on hearing. Continuous sound exposure (e.g. shipping) mostly contributed to the stress response cascade (Fig. 2; see Box 4). In the sections below, we provide an overview of these effects for each anthropogenic noise category, with references to sound levels used in studies of the impact of impulsive sound, but more detailed summaries of sound exposure metrics and documented responses for experimental studies of the impact of sound on marine life referenced in this Review are provided in Table S1.

Box 2. Sound exposure terminology Intensity of a source is reported as the source level (SL) at 1 m distance from the source (dB re. 1 µPa m). Sound exposure level (SEL) is a metric to quantify the overall acoustic energy over time, which is particularly useful when considering cumulative impacts (Erbe, 2011; Popper and Hastings, 2009; Southall et al., 2019). Thus, unlike SL or SPL, SEL is a measure of signal energy that also considers the duration of exposure. The unit of SEL is dB re. 1 µPa^2^ s. There is no accepted standard for the duration over which the energy is summed, hence that duration should be specified. If SEL is calculated for a single pulse or signal, for example in pile driving studies, it is referred to as single-strike SEL (SEL_ss_). If it is computed for multiple pulses or signals, it is cumulative sound exposure level (SEL_cum_). Exposure measurement can be weighted by the assumed hearing response of the listener. Our knowledge of the hearing response varies across taxa, but theoretical auditory weighting curves have been developed for different marine mammal functional hearing groups (Houser et al., 2017). SELs calculated using different weighting curves cannot be directly compared.

## Marine traffic

Marine traffic, comprising merchant vessels (tankers, cargo ships, etc.), cruise ships, military vessels, research vessels, fishing boats, ferries and personal watercraft, causes a substantial increase in ocean ambient sound levels (Hildebrand, 2009). Merchant vessels contribute to persistent, elevated sound levels over ocean basins, especially at frequencies below 200 Hz (McDonald et al., 2006; Ross, 2005; Širović et al., 2016; ZoBell et al., 2024), but they also substantially increase levels across broad frequency ranges in their immediate vicinity (Hermannsen et al., 2014). Smaller vessels emit broader bandwidth, lower intensity sound (source levels 140–166 dB versus approximately 195 dB re. 1 μPa m for large vessels), resulting in shorter propagation ranges (Erbe, 2013; Gassmann et al., 2017; Parsons and Meekan, 2020). In addition to physiological impacts from vessel noise addressed here, ships can also cause blunt trauma to aquatic life during direct interactions with propellers, hulls and anchors (Lightsey et al., 2006; O'Shea, 1995; Rockwood et al., 2020).

### Invertebrates

Shipping sound has physiological effects on various invertebrate taxa. Several crustacean species exhibit an increased stress response when exposed to shipping sound. Stress was apparent from increased levels of heat-shock protein (HSP)27 and HSP70 in the brain of common prawns (*Palaemon serratus*) (Filiciotto et al., 2016) and spiny lobsters (*Palinurus elephas*) (Filiciotto et al., 2014), reduced DNA integrity of common prawns (Filiciotto et al., 2016), or increased oxygen consumption of shore crabs (*Carcinus maenas*) (Wale et al., 2013). Shipping sound also reduced the number and survival of offspring. Key crabs (*Neohelice granulate*) showed negative effects on offspring survival (Moyano et al., 2024). Females of a rotifer, *Brachionus plicatilis*, produced fewer and smaller eggs when exposed to boat playback (Aspirault et al., 2023). A field study on the impact of boat sounds on the early life of sea hares (*Stylocheilus striatus*) found reduced egg development and increased mortality in veligers exposed to playback (Nedelec et al., 2014).

However, not all studies found effects of shipping sound. Solan et al. (2016) used artificial broadband noise, that was intended to represent offshore shipping to investigate its effects on the behavior and physiology of Manila clams (*Ruditapes philippinarum*), brittle stars (*Amphiura filiformis*) and Norway lobster (*Nephrops norvegicus*) and detected no physiological changes.

### Fishes

The response to sound in fishes depends on their hearing ability; fishes with specialized hearing adaptations are more vulnerable to hearing impairment than non-specialized fishes (*sensu* Popper et al., 2021). Studies using either the playback of boat sound or other continuous sound (e.g. white noise) have shown that a temporary threshold shift (TTS; see Box 3) due to hair cell loss can occur in pressure-sensitive freshwater species, such as fathead minnows (*Pimephales promelas*), goldfish (*Carassius auratus*), catfish (*Pimelodus pictus*) and zebrafish (*Danio rerio*), followed by recovery within a few days (Amoser and Ladich, 2003; Breitzler et al., 2020; Scholik and Yan, 2002b; Smith et al., 2006). In contrast, fish species lacking hearing specializations, such as the marine bluegill sunfish (*Lepomis macrochirus*) do not exhibit TTS in response to long-term sound exposure (Scholik and Yan, 2002a). Permanent threshold shift (PTS; see Box 3) or mortality due to ship sound has not been reported in fish (Popper and Hawkins, 2019; Popper et al., 2014).

Box 3. Hearing loss terminology Extensive research has been conducted on hearing loss in humans (Prell, 2012) and the same concepts and terminology apply to animals (Au, 2000; Fay and Popper, 1999). Hearing impairment, resulting from exposure to very intense signals or prolonged exposure to less intense signals, can be temporary or permanent. Such an exposure will result in an increase of the threshold at which the sound can be detected by an organism. Often, this impairment will affect only one part of the hearing range. If this impairment is temporary, it is called a temporary threshold shift (TTS), and if it is permanent, it is considered a permanent threshold shift (PTS). The severity of TTS generally increases with noise sound pressure level (SPL) and duration of sound exposure (Finneran, 2015; Prell, 2012). In the case of TTS, depending on its severity and type of sound exposure, the time until recovery to pre-exposure levels ranges from minutes to hours or sometimes days or weeks. TTS occurs as a result of recoverable inflammation and apoptosis in several sensorineural inner ear structures in response to the experienced trauma (Ryan et al., 2016). In contrast, PTS, particularly due to impulsive and intense sounds, can result from damage to the tympanic membrane of the outer ear, or dislocation of ossicular bones in the middle ear in marine mammals. However, generally, PTS is associated with damage and loss of hair cells in the inner ear and as such only occurs in marine mammals, while fish and invertebrates can mostly regenerate those sensory hair cells (Popper and Hawkins, 2019). The damage in mammals is probably not direct mechanical damage but related to the generation of reactive oxygen species within hair cells (Kujawa and Liberman, 2009), which leads to an activation of stress signaling pathways and ultimately cell damage, apoptosis or necrosis (Ryan et al., 2016).

Many studies have reported physiological effects from boating and shipping sounds in fishes with a wide range of hearing sensitivities, such as increased cortisol levels (Wysocki, 2006) or elevated metabolic, ventilation and heart rates (Jain-Schlaepfer et al., 2018; Simpson et al., 2015). The stress response may depend on overall ship sound levels as well as the intermittency and lack of predictability of the sound, as shown in captive giant kelpfish (*Heterostichus rostratus*) (Nichols et al., 2015).

A long-term playback experiment of different boat noises elicited increased plasma levels of adrenocorticotropic hormone, glucose, lactate and hematocrit in gilthead sea bream (*Sparus aurata*) (Celi et al., 2016). Lusitanian toadfish (*Halobatrachus didactylus*) breeding males exposed *in situ* to boat sound playback for up to 2 weeks showed higher cortisol levels and a decrease in the activity levels of energy metabolism-related biomarkers, accompanied by a lower reproductive outcome (Amorim et al., 2022). These results contrast with the field study of juvenile three spot damselfish (*Dascyllus trimaculatus*), which habituated to boat sound playback after 2–3 weeks, no longer showing elevated cortisol levels and ventilation rates (Nedelec et al., 2016).

Box 4. The stress response cascade **Primary response**

The primary stress response is initiated by the neuroendocrine system. When an animal perceives a stressor, e.g. a predator or a toxin, the central nervous system signals the release of key hormones. In fish, this involves the hypothalamic–pituitary–interrenal (HPI) axis, which is analogous to the hypothalamic–pituitary–adrenal (HPA) axis in mammals (Pottinger, 2008). The HPI/A axis regulates hormone excretion via secretion of corticotropin-releasing hormone (CRH) (Smith and Vale, 2022), culminating in the release of catecholamines (such as adrenaline) and corticosteroids (such as cortisol) (Di Cosmo and Polese, 2016; Kennedy and Janz, 2023; Pottinger, 2008). Invertebrates do not possess a HPA/I axis, but identification of a CRH binding protein ortholog signified the conservation of the stress signal pathway across taxa (Ketchesin et al., 2017).

**Secondary response**

The secondary stress response consists of the physiological changes triggered by the primary hormonal release. These changes are designed to help the animal cope with the stressor. Key secondary responses in marine animals include: (i) metabolic changes: increased levels of blood glucose and lactate provide a quick energy source for the muscles; (ii) hydromineral balance: in fish, stress can increase gill permeability to water and ions, disrupting their internal salt and water balance. Cortisol plays a critical role in restoring this balance (Kennedy and Janz, 2023); (iii) cardiovascular and respiratory changes: heart rate and blood pressure changes affect oxygen delivery to tissues; they increase under stress in fishes and decrease in diving marine mammals (Atkinson et al., 2015); and (iv) immune system modulation: short-term stress may boost some immune functions, but prolonged or chronic stress can suppress the immune system (Di Cosmo and Polese, 2016; Kennedy and Janz, 2023; Martyniuk et al., 2025).

**Tertiary response**

Tertiary responses are the long-term, whole-animal effects of chronic stress. If the stressor is severe or prolonged, the initial adaptive responses can become maladaptive, leading to negative consequences for the animal's health and survival (Di Cosmo and Polese, 2016; Kennedy and Janz, 2023; Martyniuk et al., 2025; Pottinger, 2008). These include: (i) reduced growth: energy is diverted from growth to coping with the stressor; (ii) impaired reproduction: stress can disrupt reproductive hormones and behaviors; (iii) decreased disease resistance: a weakened immune system leads to a higher risk of illness.

Physiological responses to stress can vary across marine species as a result of their unique adaptations to the environment. For instance, marine mammals, which have adaptations to manage nitrogen loads during deep dives, may experience decompression sickness as a result of their behavioral response to stress, which can disrupt their normal gas exchange mechanisms (Atkinson et al., 2015; Cox et al., 2006; Fahlman, 2024).

Playback of boat sound (or other continuous sound) in both *in situ* and laboratory experiments with early-life stages indicates physiological stress responses that may have carry-over effects to later stages. It may lead to increased cortisol levels and heart rate, and altered biochemical stress responses, assessed by oxidative stress and energy metabolism biomarkers (Fakan and McCormick, 2019; Faria et al., 2022; Jain-Schlaepfer et al., 2018; Lara and Vasconcelos, 2021). Boat sound exposure may also impact development and survival rates but there are mixed, species-specific results, ranging from no effects to variability in development outcomes, and increased mortality (Bruintjes and Radford, 2014; Fakan and McCormick, 2019; Faria et al., 2022; Lara and Vasconcelos, 2021).

### Marine mammals

Physiological effects from shipping sounds have not been extensively documented for marine mammals. A study of killer whale (*Orcinus orca*) responses to shipping sounds indicated a change in respiration rate in addition to the observed behavioral responses, but no direct measure of the magnitude of that change was reported and the response may have been from the physical presence of boats, not their sound field (Williams et al., 2014).

## Seismic exploration

The oil and gas industry relies on sub-seafloor acoustic imaging, accomplished with air-guns or water-guns, to develop profiles of the Earth's crust below the seafloor. Arrays of air-guns are typically towed behind ships, focusing the signal vertically. Seismic exploration sounds can dominate basin-wide soundscapes, as a result of their high intensity and low frequency, for periods of weeks to months (Nieukirk et al., 2004; Širović and Holcer, 2021; Wiggins et al., 2016). Seismic surveys for research purposes, conducted in areas not subject to commercial surveys, are less common. In addition, ‘light’ seismic surveys are at times conducted in coastal areas before infrastructure construction projects (Gontz et al., 2006).

### Invertebrates

Seismic exploration has a negative reputation in the invertebrate fishing industry; hence, the physiological effects of seismic surveys have been studied for a number of invertebrate taxa (Carroll et al., 2017). However, the results are mixed. Some studies indicated increases in mortality in the scallop *Pecten fumatus*, and a variety of zooplankton including cladocerans, euphausiids, appendicularians, decapods, polychaetes and mollusks, possibly lasting months after the seismic air-gun survey at sound exposure levels (SELs; see Box 2) ranging between 153 and 221 dB re. 1 μPa^2^ s (Day et al., 2017; Fields et al., 2019; McCauley et al., 2017), while others did not find significant effects in Acroporidae and Agaricidae coral families, pearl oyster (*Pinctada maxima*) or three species of scallops across comparable SEL ranges (Heyward et al., 2018; Parsons et al., 2024; Przeslawski et al., 2018).

When evaluating sub-lethal effects of seismic surveys, rock lobsters (*Jasus edwardsii*) experienced long-term damage to sensory hairs of statocysts and increased time between molts at SELs around 190 dB re. 1 μPa^2^ s (Day et al., 2019, 2020, 2022), New Zealand scallop (*Pecten novaezelandiae*) larvae experienced delays and malformations in development at SEL 161–165 dB re. 1 μPa^2^ s (Aguilar De Soto et al., 2013) and scallop *Pecten fumatus* experienced changes in hemocyte density at cumulative SELs of approximately 190 dB re. 1 μPa^2^ s (Day et al., 2017). In contrast, Acroporid and Agaricid corals showed no skeletal damage after exposure to SEL 204 dB re. 1 μPa^2^ s (Heyward et al., 2018).

Partly, these differences are due to small sample sizes and the lack of sufficient controls (Carroll et al., 2017), but the reason for the varying results could also be the variety of species examined. Even for one of the few species that has been studied repeatedly, the snow crab (*Chionoecetes opilio*), the impacts of seismic survey sound showed a lot of variability, indicating that the differing results may be related to stochastic processes rather than sound impact (Morris et al., 2020).

### Fishes

Similarly, the presumed negative impact of seismic surveys on fisheries has been driving demand for scientific knowledge in this field for fish species (Meekan et al., 2021; Sivle et al., 2021). No mortalities have been reported in studies addressing the effects of exposure of adult fish to seismic air-guns (Hawkins and Popper, 2018; Popper et al., 2005). However, some larval fish can experience mortality and physical injuries when exposed to seismic surveys, but only when within a few (<5) meters of the air-guns, while others have shown no effects (Carroll et al., 2017; Sivle et al., 2021).

When it comes to hearing, some fish species, such as northern pike (*Esox lucius*) and lake chub (*Couesius plumbeus*) experienced temporary hearing loss but no sensory cell damage at air-gun sound exposure with SEL 178 dB re. 1 μPa^2^ s, while broad whitefish (*Coregonus nasus*) experienced neither at similar SELs (Popper et al., 2005; Song et al., 2008). In contrast, when pink snapper (*Pagrus auratus*) were exposed to air-guns at a sound pressure level (SPL; see Box 1) of 203 dB_rms_ re. 1 μPa, the sensory cells of their inner ear were extensively damaged and did not recover (McCauley et al., 2003).

Both tank-based and *in situ* studies on air-gun or air-gun-like exposure of fish point to primary and secondary stress responses, such as increased levels of adrenaline and cortisol, altered biochemical indicators and changes in heart rate or ventilation rate in Atlantic salmon (*Salmo salar*), European sea bass (*Dicentrarchus labrax*) and Atlantic cod (*Gadus morhua*) at SELs between 134 and 175 dB re. 1 μPa^2^ s (Davidsen et al., 2019; Radford et al., 2016; Santulli et al., 1999; Sverdrup et al., 1994).

### Marine mammals

Direct studies on the effect on cetacean physiology of seismic exploration have largely been limited to hearing studies in a captive environment. A harbor porpoise (*Phocoena phocoena*) experienced TTS after exposure to a single impulse SEL of 162 dB re. 1 μPa^2^ s (Lucke et al., 2009) as well as exposure to 10 and 20 consecutive air-gun shots with a cumulative sound exposure level (SEL_cum_; see Box 2) of 188 and 191 dB re. 1 μPa^2^ s (Kastelein et al., 2017). In a subsequent study with the same sound sources, the same animal showed no significant TTS, possibly as a result of self-mitigation via suppressed hearing sensitivity (Kastelein et al., 2020). A beluga whale (*Delphinapterus leucas*) showed onset of TTS at SEL 186 dB re. 1 μPa^2^ s when exposed to sounds from seismic water-guns (Finneran et al., 2002). This beluga was screened for a neural-immune response to seismic impulses as a measure of ‘stress’ and had increasing noradrenaline (norepinephrine), adrenaline (epinephrine) and dopamine levels as sound levels were raised (Romano et al., 2004). A bottlenose dolphin (*Tursiops truncatus*) in this same study did not show TTS (Finneran et al., 2002). However, another bottlenose dolphin showed TTS at 8 kHz and two had suppressed amplitudes of auditory steady-state responses at SEL_cum_ ∼180 dB re. 1 μPa^2^ s (Finneran, 2015). Spotted (*Phoca largha*), bearded (*Erignathus barbatus*) and ringed seals (*Pusa hispida*), and a California sea lion (*Zalophus californianus*) exposed to a relatively low level, single air-gun pulse did not experience TTS in a laboratory setting (Finneran et al., 2003; Reichmuth et al., 2016), but after exposure to multiple pulses with SEL_cum_ 191–195 dB re. 1 μPa^2^ s, bearded seals experienced TTS (Sills et al., 2020).

Field observations of marine mammal responses to seismic surveys are very rare, but a recent tagging study of narwhals (*Monodon monoceros*) found that two individuals exposed to low duty cycle air-gun sound exhibited prolonged periods of high-intensity swimming with elevated stroke frequencies, yet extended and intense bradycardia (<10 beats min^−1^) and increased post-dive respiratory rates (Williams et al., 2022).

## Pile driving

Pile driving is a construction activity that involves pounding stabilizing sheets or piles into the seabed using large hammers. The peak source levels of this activity are high, between 226 and 248 dB re. 1 μPa at 1 m (Bailey et al., 2014; Miller et al., 2017). Pile driving activities typically last days or weeks and occur at predetermined construction sites. Therefore, this activity is considered high impact, but geographically constrained.

### Invertebrates

Studies of pile driving have documented a variety of physiological responses in invertebrates. Pile driving sounds induced damage in the hearing organs of cuttlefish (*Sepia officinalis*) (Solé et al., 2022) but even at a SEL_cum_ up to 214 dB re. 1 μPa^2^ s did not lead to TTS in longfin squid (Jézéquel and Mooney, 2024).

Pile driving studies on mollusks mainly reported effects on secondary and tertiary stress responses. Exposure to pile driving sounds increased metabolic rate of giant sea scallops (*Placopecten magellanicus*) (Cones et al., 2024). In one study, artificial intermittent broadband sound with 150 dB re. 1 μPa^2^ s SEL was played back to Manila clams, brittle stars and Norway lobster, but there were no significant effects of sound exposure on tissue glucose, lactate concentration or glycolytic activity (Solan et al., 2016). In cuttlefish (Solé et al., 2022) and great scallop (*Pecten maximus*) (Gigot et al., 2024, 2023), exposure to pile driving noise decreased offspring survival, although it did not reduce egg laying rate in longfin squid (*Doryteuthis pealeii*) (Jones et al., 2025). However, pile driving increased growth rate in the surviving scallop larvae (Gigot et al., 2024, 2023).

### Fishes

In fishes, controlled lab experiments to evaluate injury response to pile driving have shown that pile driving can lead to severe barotrauma but rarely to mortality. Injuries were found in the gas bladder and surrounding organs such as the liver, kidney and gonads of hybrid striped bass *Morone chrysops×M. saxatilis*, and Mozambique and Nile tilapia (*Oreochromis mossambicus* and *Oreochromis niloticus*, respectively), chinook salmon (*Oncorhynchus tshawytscha*) and lake sturgeon (*Acipenser fulvescens*) exposed at a SEL_cum_ above 200 dB re. 1 μPa^2^ s as well as in the inner ear of hybrid striped bass and Mozambique tilapia exposed at similar levels (Casper et al., 2012, 2013a,b; Halvorsen et al., 2012a,c). The risk of injury was higher in fish with a closed gas bladder and in larger fish, probably because of their larger gas bladders. The extent of tissue damage was linked with the cumulative level of sound exposure, with damage to tissues and organs preceding damage in the inner ear (Casper et al., 2017; Halvorsen et al., 2012a). Fish without a gas bladder (e.g. flatfish) presented no injuries (Halvorsen et al., 2012c). Very little data on mortality are available, and it only seems to occur when fish are very close to pile driving sources (Popper and Hawkins, 2019). No data are available on TTS for fish exposed to pile driving (Popper and Hawkins, 2019).

Pile driving noise at lower levels (SEL 184 dB re. 1 μPa^2^ s reported in Bruintjes et al., 2016b) can elicit secondary stress responses in fishes, such as elevated oxygen uptake in juvenile European seabass (*Dicentrarchus labrax*) and adult black seabream (*Spondyliosoma cantharus*) (Bruintjes et al., 2016a,b; Spiga et al., 2017), but it elicited no changes in European plaice (*Pleuronectes platessa)* adults (Bruintjes et al., 2016b), a species with no gas bladder. Interestingly, the opposite was found when juvenile European sea bass were exposed to *in situ* pile driving activity: their oxygen consumption rate and whole-body lactate concentration decreased (Debusschere et al., 2016). Exposure of Atlantic salmon (*Salmo salar*) to pile driving noise at SPL 164 dB_rms_ re. 1 μPa did not change their oxygen consumption (Harding et al., 2016).

Although planktonic larvae may suffer more from sound exposure than juveniles and adults, as a consequence of their limited ability to avoid it, only a few studies assessed the impacts of pile driving on this early life stage. In controlled exposure experiments with SEL_cum_ 206 dB re. 1 μPa^2^ s, common sole (*Solea solea*), European sea bass and Atlantic herring (*Clupea harengus*) larvae showed no increased mortality compared with control groups across larval stages (Bolle et al., 2012).

### Marine mammals

Research on the physiological effects of pile driving in marine mammals was driven largely by the development of offshore wind farms in the North Sea; hence, the primary species targeted were harbor seal (*Phoca vitulina*) and harbor porpoise, two marine mammal species of concern in the region. Their only documented physiological responses to pile driving were established from laboratory playback experiments. Two harbor seals experienced TTS after prolonged exposure (3 h) to SEL_cum_ 192 dB re. 1 μPa^2^ s, but recovered within an hour (Kastelein et al., 2018). Harbor porpoises exposed to pile driving sounds with SEL_cum_ 180 dB re. 1 μPa^2^ s also showed TTS (Kastelein et al., 2015). Otherwise, most of the evidence of a response to pile driving in marine mammals is related to behavioral changes (e.g. Madsen et al., 2006; Tougaard et al., 2012).

## Sonar

Sonars, including naval, fisheries and scientific, are impulsive sources that can operate over a wide range of source levels and frequency ranges. Military sonars, which are developed and used primarily for anti-submarine warfare, tend to be high intensity (up to 235 dB re. 1 μPa at 1 m) and operate at frequencies from 100–2000 Hz (low-frequency active sonar, LFAS) to 2–8 kHz (mid-frequency active sonar, MFAS), insonifying large areas (Alves et al., 2014; Hildebrand, 2009). Non-military sonars are used for mapping of the ocean floor or the water column; they operate over 12–400 kHz and with lower source levels (Hildebrand, 2009). Their beams are more narrowly focused, resulting in a much narrower insonification area (a few hundred meters or several kilometers).

### Invertebrates

LFAS falls within the known hearing range of invertebrates. Although no studies have exposed invertebrates to LFAS specifically, cuttlefish, common squid (*Loligo vulgaris*), common octopus (*Octopus vulgaris*) and southern shortfin squid (*Illex coindetii*) exposed to 50–400 Hz sinusoidal wave sweeps with SPL 157 dB re. 1 μPa exhibited substantial lesions and damage to sensory epithelia, ruptured membranes and nerve damage (André et al., 2011). Additionally, common cuttlefish exposed to 100–400 Hz frequency sweeps with SPL 139–141 dB re. 1 μPa displayed damaged hair cells directly and 48 h after exposure, an effect that decreased with distance from the sound source (Solé et al., 2017). Also, two medusa species, *Cotylorhiza tuberculata* and *Rhizostoma pulmo*, exposed to similar low-frequency (50–400 Hz) sweeps at SPL 157 dB re. 1 μPa experienced acoustic trauma to their statocyst sensory epithelium (Solé et al., 2016).

Invertebrates are generally not known to be sensitive to sounds >5 kHz, and therefore are unlikely to be heavily impacted by higher frequency disturbances such as MFAS (Duarte et al., 2021). One study of sonar impacts, in which sonar-like signals at SPL 177–182 dB re. 1 μPa were coupled with lower frequency shipping noise, indicated a delayed increase in hemolymph glucose for juvenile blue crabs (*Callinectes sapidus*) and American lobsters (*Homarus americanus*), but showed no other physiological responses (Hudson et al., 2022).

### Fishes

Studies on the impact of sonar on fish physiology are limited. The impact of LFAS has been investigated on rainbow trout (*Oncorhynchus mykiss*), yellow perch (*Perca flavescens*), largemouth bass (*Micropterus nigricans*) and channel catfish (*Ictalurus punctatus*) with a small TTS documented only in channel catfish at an SPL of approximately 195 dB re. 1 μPa (Halvorsen et al., 2013; Popper et al., 2007). Exposure to MFAS at a much higher SPL of 210 dB re. 1 μPa had no effect on rainbow trout, but it caused TTS lasting less than 24 h in some channel catfish exposed to these sounds (Halvorsen et al., 2012b). No physiological damage or mortalities were associated with exposure to either military sonar type for these species (Halvorsen et al., 2012b; Kane et al., 2010; Popper et al., 2007). Likewise, no tissue damage or significant mortality resulting from MFAS has been found in eggs and larvae of different fish species (Popper and Hastings, 2009).

### Marine mammals

MFAS was first demonstrated to be lethal to goose-beaked whales (*Ziphius cavirostris*), Blainville's beaked whales (*Mesoplodon densirostris*) and northern minke whales (*Balaenoptera acutorostrata*) during a mass stranding in the Bahamas in 2000 (Balcomb and Claridge, 2001). Several beaked whale strandings resulting in mortality related to naval sonar have occurred since (Filadelfo et al., 2009; Parsons, 2017; Simonis et al., 2020). Other cetacean species whose mortality occurred coincidentally with naval sonar exercises include dwarf sperm whales (*Kogia sima*), short-finned pilot whales (*Globicephala macrorhynchus*), long-finned pilot whales (*Globicephala melas*) and common dolphins (*Delphinus delphis* and *Delphinus capensis*), with deaths of pygmy sperm whale (*Kogia breviceps*) and three additional species of delphinids also possibly related to sonar activity (Parsons, 2017).

Multiple studies have been conducted on the effect of sonar on captive cetaceans and pinnipeds, particularly regarding hearing impacts (Finneran, 2015). Generally, experiments to document PTS in marine mammals are not conducted because of ethical considerations, but one documented case of PTS in a marine mammal occurred in a harbor seal that was exposed to a 4.1 kHz tone at SEL 191 dB re. 1 μPa^2^ s (Reichmuth et al., 2019). Beluga whale and bottlenose dolphins experienced temporary hearing loss after repeated exposure to playback or simulated MFAS pings (Mooney et al., 2009; Schlundt et al., 2000). Killer whales exhibited masking effects when exposed to a signal meant to simulate continuous active sonar (Branstetter et al., 2024). In harbor porpoises, SEL_cum_ and an inter-pulse interval of a 1–2 kHz sonar signal were important factors in determining the strength of TTS (Kastelein et al., 2014). California sea lions in tanks suffered from TTS after exposure to MFAS frequencies, with exposure level resulting in the onset of TTS depending on frequency and playback duty cycle (Kastelein et al., 2021).

When it comes to stress response cascades, in bottlenose dolphins there was no consistent relationship between cortisol or adrenaline and received SPL during exposure to simulated MFAS (Houser et al., 2020). Hooded seals (*Cystophora cristata*) kept in an ocean net-cage showed increased heart rate in response to low- and mid-frequency naval sonar (Kvadsheim et al., 2010). No experiments on the physiological response to sonars have been conducted on free-ranging cetaceans because of the inherent challenges of such studies.

## Other industrial noise

The main contributors of other industrial noise in the aquatic environment include coastal activities such as dredging, port activities, coastal development construction, aquaculture and shipyard activities, as well as the introduction and operation of energy-producing installations that can occur both coastally and offshore. Generally, these sound sources have most of their energy at low frequencies (20 Hz to 1 kHz) and are of long duration, and are thus generally considered continuous noise sources, but may also include impulsive sources such as pile driving, which we considered separately, and explosions.

### Invertebrates

There are few studies on the effect of other industrial noise on invertebrates. Exposure to high levels of drilling sounds resulted in decreased survival and damage to hearing organs in cuttlefish (Solé et al., 2022) and decreased survival of great scallop larvae (Gigot et al., 2023). Whiteleg shrimp (*Litopenaeus vanname*) exposed to recordings of recirculating aquaculture system sounds did not exhibit any stress-related effects (Slater et al., 2020). While we identified few dedicated studies, further effects of other industrial noise sources on invertebrate physiology are not improbable, as their low frequencies strongly overlap with the sensitive hearing range of invertebrates.

### Fishes

Several studies on the impact of explosives on fish reported lethal impacts in fish exposed to blasts; the risk of injury and mortality is higher in fish with gas bladders as a consequence of barotrauma (Popper and Hastings, 2009). Other studies point to damage of both non-auditory tissues and the inner ear in Pacific sardine (*Sardinops sagax*) and Pacific mackerel (*Scomber japonicus*) (Dahl et al., 2020; Jenkins et al., 2022; Smith et al., 2022). A study of larval and small juvenile pinfish (*Lagodon rhomboides*) and spot (*Leiostomus xanthurus*) found similar damage to non-auditory tissues but also indicated they seem to be more vulnerable to underwater shock waves than larger juveniles and adult fishes (Govoni et al., 2008).

Otherwise, studies on the impacts of aquaculture noise found that Atlantic cod had a lower spawning performance, probably associated with elevated egg cortisol levels (Sierra-Flores et al., 2015), Atlantic salmon (1+ post-smolt) exhibited an increase in plasma cortisol and a decrease in neuronal activity (Oppedal et al., 2025), but Atlantic salmon parr (Slater et al., 2020) and rainbow trout did not show a response (Wysocki et al., 2007). General white noise exposure appeared to affect liver metabolism in a hybrid sturgeon (*Acipenser baerii*×*A. schrencki*) (Zhang et al., 2022). A larger diversity of studied species will be necessary for a better understanding of the effects of underwater explosions and other industrial noise on fishes (see Jenkins et al., 2022, for suggestions of future direction).

### Marine mammals

No direct studies on the physiological impact of other industrial noise on marine mammals were identified in this Review. However, there is some evidence marine mammals can sustain ear damage when found in the vicinity of explosions. In one case, humpback whales (*Megaptera novaeangliae*) caught in fishing nets off Newfoundland had ear damage consistent with blast injuries (Ketten et al., 1993). In another, half of the Weddell seals (*Leptonychotes weddellii*) sampled after dynamite explosions in McMurdo Sound had ear tissue damage (Bohne et al., 1986; Bohne et al., 1985). Finally, juvenile harbor porpoises showed inconclusive metabolic effects when exposed to pings from acoustic harassment devices (Elmegaard et al., 2023).

## Discussion

**Fig. 3.**
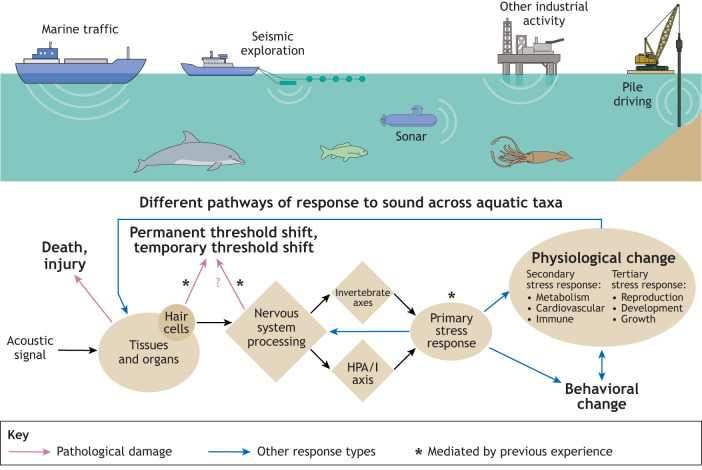
**Schematic diagram of sound sources described in this study and different pathways (black arrows) of response to sound across marine taxa.** Responses to sound comprise: death, injury, physiological change and behavioral change. Pink arrows indicate pathological damage and blue arrows indicate other response types; asterisks indicate mediation of responses by previous experience. Diamonds indicate points with substantial taxonomic differences. Note that ‘Invertebrate axes’ could in fact represent multiple different mechanisms for stress responses in invertebrates.

Studies on the impact of exposure to ship sounds and seismic surveys were the most common in our review of the literature (Fig. 1). In most studies on fishes and invertebrates, the presence of ship sounds led to increased stress or indicators of stress. While it could be argued that the number of studies is not sufficient to cover the breadth of taxonomic diversity, given the similarity of the response for a broad range of organisms, this could indicate a common underlying mechanism for this impact (Fig. 3).

Findings from studies on other sound sources show mixed results across taxa. The most severe documented physiological impacts were from naval sonar on marine mammal species. It has been proposed that morbidity in the case of MFAS can occur both from the initial primary tissue damage and from decompression sickness resulting from the behavioral response (Cox et al., 2006). Fish and invertebrates, conversely, may show some hearing damage due to LFAS.

Effects of exposure to seismic noise and pile driving varied across taxa and exposure conditions. In a few studies conducted on invertebrates, impact from air-guns ranged from severe to none. Studied fishes more often exhibited physiological responses to those sounds than not, with potential for injury from nearby pile driving activities, while studied marine mammals often experienced hearing impairment after exposure. Currently, impacts from other industrial noise sources are poorly documented and uneven across taxa. Some information points to the potential for injurious or lethal effects of explosions on some marine mammals and fishes, but less impact from continuous sound sources. For fish and invertebrates, responses generally appear to be species and developmental-stage specific.

Many gaps remain in our understanding of the physiological impacts of anthropogenic sounds on aquatic life. While marine mammals dominate the number of studies conducted when accounting for behavioral impact studies (Popper et al., 2020), when considering only physiology, and especially if looking beyond hearing impacts, studies on fishes were more common in our review of the literature (Fig. 2). As mentioned previously, however, a limited number of species has been used with very varied stimuli, making it challenging to make comparisons. And while physiological impacts should not be considered in isolation from behavioral ones, lessons learned for each taxon and gaps identified across the topics should be used to enhance our knowledge and optimize efforts in the future. Below we outline four areas where more synergistic and strategic efforts could be most valuable, and which we believe deserve more research focus in the future.

### Conducting more comprehensive data collection efforts

Most studies of sound impact on physiology focus on either hearing or stress-related indicators. However, it would be relatively easy to collect multiple types of data from captive animals, which would help to elucidate mechanistic pathways of response. For marine mammal species in particular, studies were often focused solely on hearing impacts, but when similar studies in captivity are conducted on fishes, other physiological metrics, e.g. heart rate, cortisol or other hormone levels, were more commonly sampled. Considering that captive experiments already require a very large effort, collecting such physiological metrics on marine mammals would be a relatively small add-on that could yield substantial improvement in our understanding of impacts. Investigations into the molecular and cellular level effects, including protein and gene expression changes resulting from noise impacts (El-Dairi et al., 2024; Kurabi et al., 2017), would also be an important future expansion that needs to be coupled with a robust acoustic playback design. In addition, advances in technology are leading to tags that can measure heart rate and hormone levels (Aoki et al., 2021; Hvas et al., 2020), which could also enable collection of more physiological data in the field and enable comparisons with lab experiments (see ‘Ensuring comparability of data collected in the field and in the lab’, below). Combining multiple matrices to measure sound impact would help bring us closer to a more mechanistic understanding of the impacts that anthropogenic sounds have on aquatic life.

### Providing functional models for extrapolation from a model species to the species of interest

Improvements in our understanding of the underlying response mechanisms will help generalize results, but a framework for applying these mechanistic interpretations across a variety of taxa is still needed. Within such a framework, standardized measures would be employed to assess whether the assumptions of the model are appropriate for the species of interest. Popper and colleagues (2014) recommended a framework to analyze sound exposure risk in fish based on the absence or presence of gas chambers and the potential of those structures to enhance hearing. That approach allows for extension of information from a limited number of species to others with similar hearing physiology in the absence of other data. In the case of fish and invertebrates, it might also be important to have separate models for larval stages when they are functionally different from adults (Carroll et al., 2017; Popper et al., 2014). For marine mammals, categorization is based on hearing frequency ranges and developed weighting curves (Houser et al., 2017; Southall et al., 2007). Thus, low-frequency marine mammals (i.e. baleen whales) are grouped together and presumed to have similar hearing abilities, as are high-frequency ones (i.e. odontocetes). However, there are clear differences within those groups; for example, beaked whales appear to be far more susceptible to MFAS than other odontocetes. Even in the case of species that have been well studied, such as harbor porpoises and harbor seals, developed guidelines for the onset of TTS do not seem always to be good predictors of actual TTS onset (Tougaard et al., 2022). This could indicate there are additional mechanisms in play that mediate or inhibit the acoustic response (Kastelein et al., 2020), as has been found in other mammals (Koch and Schnitzler, 1997). A framework for the categorization of invertebrates is currently lacking but may be particularly important considering their vast diversity. They may be the appropriate group to approach with the mechanistic functioning framework, using hearing abilities and response mechanisms to categorize groups that may exhibit similar responses. To accomplish that, however, mechanisms of response to sound need to be tested across a broad set of species, encompassing various invertebrate phyla.

### Ensuring comparability of data collected in the field and in the lab

There has been a healthy debate in the bioacoustics community regarding the applicability of findings from tank and other captive impact studies to real-world situations. Lab conditions allow for easier data collection and more straight-forward result interpretation. They also provide the possibility to disentangle interacting mechanisms of disturbance by changing one environmental parameter at a time. Conversely, they raise questions of how representative those results are for animals in the wild. It can be difficult to re-create sound pressure and particle motion fields for signals of interest in a tank (Akamatsu et al., 2002) to elicit a response representative of responses in the wild (Hawkins and Popper, 2017). Similarly, rearing animals in a tank possibly exposes them to a high-noise environment throughout their development which could affect their response to noise exposure (Fig. 3). Moreover, testing animals in captivity removes the possibility of escaping from the sound source, an option available in the wild, which can induce stress (Popper and Hawkins, 2019). An additional problem with marine mammal captive studies is that most exposure and impact tests are conducted on a small sample of animals. Often, the same individual may be subjected to repeated tests (Finneran, 2015). Realistically, captive experiments will have to continue to be part of our efforts to make progress in this area. Hence, we need to improve our understanding of how results from captive environments translate to wild conditions (Neo et al., 2016; Radford et al., 2016). This may be achieved by collecting the same types of physiological metrics in lab and field conditions for direct comparison in species where this is feasible or by devising well-replicated captive experiments that are generalizable to the wild in cases where wild experimentation is challenging. Avoiding pseudoreplication by using multiple animals, employing real-world sound fields in playback experiments and conducting mesocosm experiments are some of the ways in which this can be accomplished.

### Considering impacts of sound in a changing world

Sound is not the only stressor for aquatic life and physiological effects are just one part of the animal response. Even though the focus of this Review was on physiology, to assess the full impact, it is important to consider all the combined effects from sound, as well as other pressures that animals face. Theoretical frameworks have been developed to look at cumulative effects of multiple stressors on animal populations (Booth et al., 2020; King et al., 2015; National Academy of Sciences, 2005), but those frameworks require input of relevant data, such as reproductive success or population growth, which are rarely obtained directly from impact studies (e.g. Soudijn et al., 2020). Obtaining links from current metrics of impact to those applicable to theoretical frameworks remains a challenge but is an area that should be prioritized. Studies on the impact of sound on reproductive output and survival rates of immature individuals would be of particular value (Booth et al., 2020).

While substantial progress has been made over the past decades in our understanding of the impact of sound on aquatic life, current approaches to managing ecosystems, whether based on comprehensive risk assessment (Popper et al., 2020) or more precautionary principles (Risch et al., 2021), will require further advancements in this field. There are numerous issues on the horizon that will continue to put pressure on aquatic animals and their acoustic space. Improving our understanding of the mechanisms of impact from sound will also allow us to effectively respond to new and future noise pollutants, such as floating offshore wind farms, ultrasonic antifouling systems on ship hulls (Trickey et al., 2022) or deep seabed mining (Levin et al., 2016), and ensure their impacts are quickly and effectively considered.

## Supplementary Material

10.1242/jexbio.250800_sup1 Supplementary information
