# Supplementary material for: Physiological effects of anthropogenic sound on aquatic animals: where are we and what is next?
Source: J Exp Biol. 2026 Feb 11;229(Suppl 1):jeb250800. doi: 10.1242/jeb.250800 (PMC12952712; doi:10.1242/jeb.250800)
Supplement: Supplementary information [file jexbio-229-250800-s1.pdf]

Table S1. Summary of experimental and observational peer-reviewed studies in which some information on sound or exposure levels was provided that were considered in this review.

Sound source category	Characteristics of sound source [1]						Common species name (Latin name), if known	Taxonomic group	Sample size	Type of effect (brief description)	Type of effect (category)[2]	Field/in situ or captive/ lab study	Reference (Author, year)
	Source type or frequency range (Hz)	Source level (dB re 1 μPa)[3]	Received level/ SPL (dB)[3]	Duty cycle	SEL or SELcum (dB re 1 μPa²s)[4]	Particle motion?							
Traffic	Looped 30 s recording from small boat		129 dB (over 100-10000 Hz)			No	*Brachionus plicatilis*	Inveterbrate (rotifer)	~700	No effect on length, but smaller and fewer eggs were produced by females	Reproduction	Captive	Aspirault et al. 2023
Traffic	100-2500		75-110 dB re 1 Hz			No	Mediterranean spiny lobster (*Palinurus elephas*)	Invertebrate (crustacea)	36	Increased locomotor activities, increased HSP 70 expression	Metabolic	Captive	Filiciotto et al. 2014
Traffic	100-2500		90-105 dB re 1Hz			Yes	Common prawn (*Palaemon serratus*)	Invertebrate (crustacea)	48	Higher total protein concentrations in hemolymph and brain, lower DNA integrity, increased expression protein levels of HSP (heat shock protein) 27 and 70 in brain tissues	Metabolic	Captive	Filiciotto et al. 2016
Traffic	Motorboat recording		123-158 dBrms	50%	143 SELcum	No	Key crab (*Neohelice granulate*)	Invertebrate (crustacea)	24 females	No differences in egg weight or volume, but increase in heartrate in later developmental stages; decrease in total protein content; lipid peroxidation in embryos	Reproduction, metabolic	Captive	Moyano et al. 2024
Traffic	Broadband (real boat recordings)		75-98 dB re 1Hz across 200-3000 Hz	15% in 12 h playback		Yes	Sea hare (*Stylocheilus striatus*)	Invertebrate (mollusca)	Eggs from13 & 11 mothers	Decreased egg development and increased veliger death	Reproduction	Field	Nedelec et al. 2014

[1] We included a number of metrics that are most commonly used to characterize sound sources; however, there are vast differences in reported values across individual studies, thus empty cell indicates that given value was not reported for that study.
[2] Categories of effect that represent different stages of stress response cascade are: stress = primary stress response; metabolic = secondary stress response; and reproduction, development = tertiary stress response
[3] Source level as well as received level or sound pressure level values include markings of dBp, dBpp, and dBrms when it was reported whether the levels were measured as zero-to-peak, peak-to-peak, or root-mean-squared, respectively.
[4] SELss denotes a single-strike SEL was reported (for impulsive signals) while SELcum marks that the reported value is cumulative SEL

Journal of Experimental Biology • Supplementary information

				during 24 h									
Traffic	Continuous broadband (100-2000) sound		135-140			No	Manila clam (*Ruditapes philippinarum*), decapod crustacean (*Nephrops norvegicus*) & ophiuroid brittlestar (*Amphiura filiformis*)	Invertebrate (bivalvia, crustacea, echinoderma)	1, 2, and 10 individuals per species x 5	No effect on tissue concentration of glucose or lactate, no changes in glycolytic activity	None	Captive	Solan et al. 2016
Traffic			148-155 dBrms			No	Shore crab (*Carcinus maenas*)	Invertebrate (crustacea)	36	Increased oxygen consumption (especially heavier crabs)	Metabolic	Captive	Wale et al. 2013
Traffic	broadband		120-140 dBrms and 122-131 dBrms; 30 dB above background	Intermittentto simulate boat passages		No	Lusitanian toadfish (*Halobatrachus didactylus*)	Fish	16 males	Decreased likelihood of receiving eggs and number of live eggs, slightly higher cortisol levels. No oxidative damage.	Reproduction, stress	Field	Amorim et al. 2022
Traffic	100-2000 Hz		127 dBrms			No	*Neolamprologus pulcher*	Fish	237 and 415 eggs	Hatching success, fry survival and fry growth not affected	None	Captive	Bruintjes and Radford 2014
Traffic	broadband		103-105 dBrms (tank), 67 dB (open water)			No	European eels (*Anguilla anguilla*) & European sea bass (*Dicentrarchus labrax*)	Fish	156 eels	Increased ventilation rate (both species)	Metabolic	Captive, also open water for seabass	Bruintjes et al. 2016a
Traffic	0-48 kHz		123-136 dBrms octave bands (62.5 Hz - 16000 Hz centre freq)			No	Gilthead sea bream (*Sparus aurata*)	Fish	40	Increased plasma levels of adrenocorticotropic hormone, glucose, lactate, and hematocrit	Stress, Metabolic	Captive	Celi et al. 2016
Traffic	0-24 kHz		90-130 dB re 1 μPa^2/ Hz) (over 0-2000 Hz)	Boat passes every 5 min		Yes	Damselfishes (*Amphiprion melanopus* & *Acanthochromis polyacanthus*)	Fish	216 eggs per species	Increased heart rates in both species; longer length at hatch in Ap	Metabolic, development	Captive	Fakan & McCormick 2019
Traffic	0-20 kHz		120–140 dBrms	Intermitt.		Yes	Lusitanian toadfish (*Halobatrachus didactylus*)	Fish	hundreds	Embryos showed reduced levels of electron transport system (ETS); larvae showed higher overall stress responses, with increased levels of superoxide dismutase and DNA damage, ETS, and reduced growth	Development	Field	Faria et al. 2021

Journal of Experimental Biology • Supplementary information

Traffic	broadband (real engine)		125-132 dBrms (over 400-2000 Hz)		180	Yes	Staghorn damselfish (*Amblyglyphidodon curacao*)	Fish	> 18 embryos	Increased heart rate	Metabolic	Field	Jain-Schlaepfer et al. 2018
Traffic	100Hz-10kHz (real ship recordings)		up to 140 dBrms			Yes	Atlantic cod (*Gadus morhua*) larvae	Fish	Up to 300	Slower initial growth in treatment condition, lower body-width to length ratio	Development	Captive	Nedelec et al. 2015
Traffic	real boat recordings			45 s every 5 min, over 2-3 weeks		Yes	*Dascyllus trimaculatus*	Fish	~140 juveniles	No increase in blood cortisol levels or difference in growth after long exposure	None	Field	Nedelec et al. 2016b
Traffic	100-2000		126-142 dBrms			No	Giant kelpfish (*Heterostichus rostratus*)	Fish	62	Acute increase in cortisol when exposed to intermittent noise, but not continuous noise	Stress	Captive	Nichols et al. 2015
Traffic	0.3-6 kHz (real engine recording)		142 dBrms			No	Fathead minnow (*Pimephales promelas*)	Fish	6	Elevated auditory threshold	Hearing	Captive	Scholik & Yan 2002a
Traffic	0-22 kHz (real ship recordings)		148 dBrms			No	European eels (*Anguilla anguilla*)	Fish	72 glass-stage eels	Elevated ventilation & metabolic rates	Metabolic	Both field and lab	Simpson et al. 2015
Traffic	0-22 kHz		156			No	Common carp (*Cyprinus carpio*), gudgeon (*Gobio gobio*) & European perch (*Perca fluviatilis*)	Fish	12 Cc, 14 Gg, 14 Pf	Elevated cortisol levels	Stress	Captive	Wysocki et al. 2006
Traffic	10-2500		105-150 dBrms			No	Killer whale (*Orcinus orca*)	Marine mammal	42 tracks	Initially minor change in respiration, then moderate change in respiration associated with increased swim speed and change in direction	Metabolic	Field	Williams et al. 2014
Seismics	real seismic pulses		160-164 dBrms		161-165 SEL	Yes	New Zealand scallop (*Pecten novaezelandiae*)	Invertebrate (bivalvia)	4881larvae total	Delay and malformations in development	Development	Captive	Aguilar de Soto et al. 2013
Seismics		191-213 dBpp		one shot per 11.6s	181-188/ 189-198	No	Scallop (*Pecten fumatus*)	Invertebrate (bivalvia)	60	Mortality, changes in hemocyte	Death, metabolic	Field	Day et al. 2017
Seismics	real seismic survey	223-227 dBpp		one shot per 11.6s	186-191/ 192-195	No, only ground motion	Rock lobster (*Jasus edwardsii*)	Invertebrate (crustacea)	8-21	Damage to sensory hairs of statocyst	Hearing	Field	Day et al. 2019
Seismics	real seismic survey		max 209-212 dBpp		max 186-190, 192-199 SELcum	Yes	Rock lobster (*Jasus edwardsii*)	Invertebrate (crustacea)	3 groups of 20	No increase in hair cell damage in previously exposed individuals	None	Field	Day et al. 2020

Seismics	real seismic survey		150-210 dBpp		~105-190	Yes	Rock lobster (*Jasus edwardsii*)	Invertebrate (crustacea)	56 juveniles & 16 puerulus	No change in mortality, some righting impairment and increased intermoult duration	Metabolic	Field	Day et al. 2022
Seismics	two airguns				183 & 221	No	Copepod (*Calanus finmarchicus*)	Invertebrate (crustacea)	3 bags of 60 individuals and 3 bags of 400 individuals per location	Mortality close to source, no sublethal effects	Death	Field	Fields et al. 2019
Seismics	0-500		226 dBp		204	No	Coral, families Agaricidae and Acroporidae	Invertebrate (cnidaria)	8 sites	No effect on mortality, skeletal damage or visible stress	None	Field	Heyward et al. 2018
Seismics	real seismic survey		178-183 dBpp		153-156	No	Zooplankton: copepods, cladocerans, euphausiidae, appendicularians, decapods, polychaetes, molluscs	Invertebrate (crustacea, mollusca)	7 control & 5 exposure net tows	Mortality	Death	Field	McCauley et al. 2017
Seismics	real seismic survey				190	No	Snowcrab (*Chionoecetes opilio*)	Invertebrate (crustacea)		No effect on catch rates	None	Field	Morris et al. 2020
Seismics	real seismic survey				169-209 SELcum	No	Silverlip oyster (*Pinctada maxima*)	Invertebrate (bivalvia)	11000	Mortality higher in only two out of 16 groups	None	Field	Parsons et al. 2024
Seismics	real seismic survey				170	Yes	Scallops (*Pecten fumatus* & *Mimachlamys asperrima*)	Invertebrate (bivalvia)	5-8 dredges per treatment period	No effect on mortality	None	Field	Przeslawski et al. 2018
Seismics	one airgun				134-175 SELcum	Yes	Atlantic cod (*Gadus morhua*) & saithe (*Pollachius virens*)	Fish	20 Gm, 9 Pv	Bradycardia in Gm	Metabolic	Field (sea cage)	Davidsen et al. 2019
Seismics	20-1000	222 dBpp; 203 dBrms	145-190 dBrms			No	Pink snapper (*Pagrus auratus*)	Fish	14	Extensive hair cell damage	Hearing	Field (cage)	McCauley et al. 2003
Seismics	real airguns		197 dBrms		178	Yes	Northern pike (*Esox lucius*), lake chub (*Couesius plumbeus*) & broad whitefish (*Coregonus nasus*)	Fish	10-20 per species	TTS in El & Cp; no effect in Cn	Hearing	Field (pentrap)	Popper et al. 2005
Seismics	< 2000		132 dBrms (< 1kHz)	50%	143	No	European seabass (*Dicentrarchus labrax*)	Fish	hundreds	Increased opercular beat rate for post-larval fish	Metabolic	Captive	Radford et al. 2016

Journal of Experimental Biology • Supplementary information

Seismics		~220 dB				Yes, but not reported	European sea bass (*Dicentratchus labrax*)	Fish	~300	Variations of cortisol, glucose, lactate, AMP, ADP, ATP and cAMP in different tissues	Stress, metabolic	Field (sea cage)	Santulli et al. 1999
Seismics	real airgun		205-209 dBp		176-180	Yes	Lake chub (*Couesius plumbeus*), northern pike (*Esox lucius*) & broad whitefish (*Coregonus nasus*)	Fish	21 Cp, 10 Cn, 29 El	No damage to the ears	None	Field (pentrap)	Song et al. 2008
Seismics	explosions simulating air guns	~246 dB		10 explosions at 7min intervals		No	Atlantic salmon (*Salmo salar*)	Fish	36	No mortality, decrease in plasma cortisol, increase in adrenaline	Stress, metabolic	Captive	Sverdrup et al. 1994
Seismics	seismic watergun				186	No	Beluga whale (*Delphinapterus leucas*) & bottlenose dolphin (*Tursiops truncatus*)	Marine mammal	1 each	TTS	Hearing	Captive	Finneran et al. 2002
Seismics	single airgun		200-212 dBpp	10 pulses	193-195	No	Bottlenose dolphin (*Tursiops truncatus*)	Marine mammal	3	Slight TTS in 1 individual at 8 kHz	Hearing	Captive	Finneran et al 2015
Seismics	two airguns			10 and 20 pulses	188 & 191	No	Harbor porpoise (*Phocoena phocoena*)	Marine mammal	1	TTS	Hearing	Captive	Kastelein et al. 2017
Seismics	four scaled-down airguns		202 dBp		max 199	No	Harbor porpoise (*Phocoena phocoena*)	Marine mammal	1	No TTS	None	Captive	Kastelein et al. 2020
Seismics	single airgun		196 dBp	single pulse	162	No	Harbor porpoise (*Phocoena phocoena*)	Marine mammal	1	TTS	Hearing	Captive	Lucke et al. 2009
Seismics	real airgun		190-207 dBpp	single shot	165-181	No	Spotted seal (*Phoca larga*) & ringed seal (*Pusa hispida*)	Marine mammal	2 each	No hearing impacts	None	Captive	Reichmuth et al. 2016
Seismics	seismic watergun		198-226 dBp			No	Beluga whale (*Delphinapterus leucas*) & bottlenose dolphin (*Tursiops truncatus*)	Marine mammal	1 each	Increased stress hormone levels	Stress	Captive	Romano et al. 2004
Seismics	30-80 (maximum energy)		190-209 dBpp		185 & 191-195	No	Bearded seal (*Erignathus barbatus*)	Marine mammal	1	No TTS at 185 SEL; TTS after exposure to multiple consecutive pulses (higher SEL)	Hearing	Captive	Sill et al. 2020
Seismics	real airgun	241 dBpp		Air-guns fired every 80 s	120-152	No	Narwhal (*Monodon monoceros*)	Marine mammal	2 exposed animals	Reduction in gliding descents and prolonged high intensity activity with elevated stroke frequencies; prolonged and intense bradycardia; increase in post-dive respiratory rates	Metabolic	Field	Williams et al. 2022

Journal of Experimental Biology • Supplementary information

Pile driving	Real pile driving			3 h of activity per day		Yes	Giant sea scallop (*Placopecten magellanicus*)	Invertebrate (bivalvia)		Reduction in mantle water oxygen levels and increase in metabolic rate	Metabolic	Field	Cones et al. 2024
Pile driving	Pile driving from real recording		148-188 dBpp	200 ms impulse repeated every 3 s			Great scallop (*Pecten maximus*)	Invertebrate (bivalvia)	Larvae	No change in mortality, but slightly increased growth	Development	Captive	Gigot et al. 2023
Pile driving	Pile driving from real recording	221		Impulse 200 ms long, repeated every 3 s		No	Great scallop (*Pecten maximus*)	Invertebrate (bivalvia)		Offspring from adults exposed to increased levels had lower survival but grew faster	Reproduction	Captive	Gigot et al. 2024
Pile driving	pile driving from real recordings		120	1 x15 min or 5 x 15-min playback	187-214 SELcum	Yes	Longfin squid (*Doryteuthis pealeii*)	Invertebrate (cephalopoda)	89 adults	No TTS	None	Captive	Jézéquel and Mooney 2024
Pile driving	30 impacts of pile driving from real recordings		>110 dB	1 min playback		Yes	Longfin squid (*Doryteuthis pealeii*)	Invertebrate (cephalopoda)	165	Egg laying occurred at similar rates in treatment and control	None	Captive	Jones et al. 2025
Pile driving	Impulsive broadband sound (100-2000)				150	No	Manila clam (*Ruditapes philippinarum*), decapod crustacean (*Nephrops norvegicus*) & ophiuroid brittlestar (*Amphiura filiformis*)	Invertebrate (bivalvia, crustacea, echinoderma)	1, 2, and 10 individuals per species x 5	No significant changes in glycolytic activity or any accumulation of tissue lactate	None	Captive	Solan et al. 2016
Pile driving	real pile driving recordings		Max 170	5 strikes, about 1 strike/sec for 4 h		No	Cuttlefish (*Sepia officinalis*)	Invetebrate (cephalopoda)	20 adults, 30 larvae	Hair cell and lateral line damage; reduction larval hatching success and increase in larval mortality	Hearing, Reproduction	Captive	Solé et al. 2022
Pile driving	broadband, 50-1000		210 dBp		206 SELcum	No	Common sole (*Solea solea*)	Fish	25 per treatment	No statistically significant change in mortality	None	Captive	Bolle et al. 2012
Pile driving			165-197 (tank), 200-202 (open water)	90%		No	European eels (*Anguilla anguilla*) & European sea bass (*Dicentrarchus labrax*)	Fish	156 Aa	Increased ventilation rate (both species)	Metabolic	Captive, also open water for seabass	Bruintjes et al. 2016a
Pile driving					184 SELcum	No	Black seabream (*Spondyliosoma cantharus*) & European plaice (*Pleuronectes platessa*)	Fish		Increased oxygen uptake for Sc, no difference in Pp	Metabolic	Field	Bruintjes et al. 2016b

Journal of Experimental Biology • Supplementary information

Pile driving					210 & 217 SELcum	No	Chinook salmon (*Oncorhynchus tshawytscha*)	Fish	228	Swim bladder hematomas, burst capillaries, intestinal hemorrhages & hematomas, hematomas of the gonads, adipose, and body musculature at higher exposure levels; intestinal and adipose hematoma at lower exposure levels	Injury	Captive	Casper et al. 2012
Pile driving					201-213 SELcum	No	Hybrid striped bass (white bass, *Morone chrysops* × striped bass, *Morone saxatilis*)	Fish	930	Ruptured swim bladder & renal hemorrhage (mortal); swim bladder herniation, swim bladder hematomas, gall bladder hemorrhage, intestinal hemorrhage, hepatic hematomas, gonadal hematomas, and adipose hematomas	Death, injury	Captive	Casper et al. 2013a
Pile driving					210-216 SELcum, 180-186 SELss	No	Hybrid striped bass (white bass, *Morone chrysops* × striped bass, *Morone saxatilis*) and Mozambique tilapia (*Oreochromis mossambicus*)	Fish	164 hybrids, 28 Om	Significant hair cell damage in hybrid exposed to highest SEL, and in only 1/11 Om	Hearing	Captive	Casper et al. 2013b
Pile driving			upto 215 dBp		191-209 SELcum	No	Hybrid striped bass (white bass, *Morone chrysops* × striped bass, *Morone saxatilis*)	Fish	384	Ruptured swim bladder and kidney hemorrhage (mortal injuries), swim bladder hematoma, herniated swim bladder	Death, injury	Captive	Casper et al. 2017
Pile driving	125-200 (dominant energy)		210-211 dBp		181-188; 215-222 SELcum	No	European sea bass (*Dicentratchus labrax*)	Fish	528	Significant reduction in oxygen consumption rate, low whole-body lactate concentrations, no changes in specific growth rate	Metabolic	Field	Debusschere et al. 2016
Pile driving	simulated airgun				204-220 SELcum	No	Chinook salmon (*Oncorhynchus tshawytscha*)	Fish	356	Barotrauma injuries from mild to mortal; 210 dB cSEL established as threshold level for tissue damage	Injury	Captive	Halvorsen et al. 2012a
Pile driving	simulated airgun				204-216 SELcum	No	Lake sturgeon (*Acipenser fulvescens*), Nile tilapia (*Oreochromis niloticus*) & hogchoker (*Trinectes maculatus*)	Fish	181 Af, 201 On, 67 Tm	Ruptured swim bladders and renal hemorrhages in *On*; hematomas and partially deflated swim bladders in *Af*; No injuries in *Tm*	Injury	Captive	Halvorsen et al. 2012b

Journal of Experimental Biology • Supplementary information

Pile driving			164 dBrms	10%		Yes	Atlantic salmon (*Salmo salar*)	Fish	24	No effect on oxygen consumption rate	None	Captive	Harding et al. 2016
Pile driving	100 - 3000		152 dBp			No	European seabass (*Dicentrarchus labrax*)	Fish	44	Higher opercula beat rate	Metabolic	Captive	Spiga et al. 2017
Pile driving	500-800			2760 strikes per h, interval 1.3 s	146 SELss; 180 SELcum	No	Harbor porpoise (*Phocoena phocoena*)	Marine mammal	1	TTS at 4 and 8 kHz	Hearing	Captive	Kastelein et al 2015
Pile driving	500-800			9.5% duty cycle	152; 192 SELcum	No	Harbor seal (*Phoca vitulina*)	Marine mammal	2 females	2-4 dB TTS only after 360 min exposure, recovery within 60 min	Hearing	Captive	Kastelein et al. 2018
Sonar	50-400 Hz sinusoidal wave sweeps		157	100%		No	Common squid (*Loligo vulgaris*), cuttlefish (*Sepia officinalis*), common octopus (*Octopus vulgaris*) & southern shortfin squid (*Illex coindetii*)	Invertebrate (cephalopoda)	5 Lv 76 So 4 Ov 2 Ic	Damaged hair cells, ejection of hair cells from epithelium; appearance of lesions increased with time after exposure	Hearing	Captive	André et al. 2011
Sonar	Simulated sonar: 1s 1.67 kHz pulse followed by 1s 2.5-4.0 kHz FM chirp, played with LF ship noise		177-182 dB	30 min in 50 min playback		Yes	Blue crabs (*Callinectes sapidus*) & American lobsters (*Homarus americanus*)	Invertebrate (crustacea)	Juveniles and sub-adults	Increase hemolymph glucose levels 7 days after exposure; no change in HSP27 levels or in mortality	Metabolic	Captive	Hudson et al. 2022
Sonar	50-400		157			No	Cnidarians *Cotylorhiza tuberculata* & *Rhizostoma pulmo*	Invertebrate (cnidaria)	8 Ct 4 Rp for exposure, 4 Ct 2 Rp for control	Hair cells ejected from sensory epithelium in Ct; rupture of plasma membrane at base of hair cells in Rp	Hearing	Captive	Solé et al. 2016
Sonar	100-400		139-141	100%		Yes	Cuttlefish (*Sepia officinalis*)	Invertebrate (cephalopoda)	18	Damaged hair cells	Hearing	Captive	Solé et al. 2017
Sonar	2800-3800		~210 dBrms	12%	220 SELcum	Yes, estimate	Rainbow trout (*Onchorhynchus mykiss*) & channel catfish (*Ictalurus punctatus*)	Fish		No effect on Om; TTS in some Ip	Hearing	Captive	Halvorsen et al. 2012c

Journal of Experimental Biology • Supplementary information

Sonar	170-320		~195 dBrms	20%	210; 215 SELcum	Yes, estimate	Largemouth bass (*Micropterus salmoides*), yellow perch (*Perca flavescens*) & channel catfish (*Ictalurus punctatus*)	Fish		No effects on hearing in Ms & Pf; small TTS in Ip	Hearing	Captive	Halvorsen et al. 2013
Sonar	170-320 & 2800-3800		193 dBrms & 210 dBrms	20% & 12% cycle		No	Rainbow trout (*Onchorhynchus mykiss*), channel catfish (*Ictalurus punctatus*) & sunfish (*Lepomis* sp.)	Fish	65 Om, 76 Ip, 34 Ls	No exposure-related pathologies	None	Captive	Kane et al. 2010
Sonar	170-320	~215	193 dBrms	16.70%	188.5	Yes, estimate	Rainbow trout (*Onchorhynchus mykiss*)	Fish	116	TTS, but no inner ear tissue damage	Hearing	Field (tanks in a lake)	Popper et al. 2007
Sonar	1-2 kHz FM upsweep		90 dB re 1 $\mu Pa^2/Hz$	19 s on/1 s off		No	Killer whale (*Orcinus orca*)	Marine mammal	2 males	Masked killer whale calls	Hearing	Captive	Branstetter et al. 2004
Sonar	600 & 3000	228 and 226				No	Cuvier's beaked whales (*Ziphius cavirostris*)	Marine mammal	multiple	Mass stranding coincidental with source use	Death	Field	Filadelfo et al. 2009
Sonar	3250-3450		115, 130, 145, 160, 175, 185			No	Bottlenose dolphin (*Tursiops truncatus*)	Marine mammal	30	No effect on stress hormone levels	None	Captive	Houser et al. 2020
Sonar	1000-2000		144-179	5-100% duty cycle	176-206 SELcum	No	Harbor porpoise (*Phocoena phocoena*)	Marine mammal	1 male	Extent of TTS depended on SELcum and inter-pulse interval	Hearing	Captive	Kastelein et al. 2014
Sonar	2 & 4 kHz, 1/6-octave noise bands		138-167 & 133-169	variable	174-203 & 196-205	No	California sea lion (*Zalophus californianus*)	Marine mammal	2	SEL for onset of TTS varied with frequencies, but no TTS at duty cycles <60%	Hearing	Captive	Kastelein et al. 2021
Sonar	1.3-1.7 kHz, 3.7-4.3 kHz, or 6.0-7.0 kHz	134-194 dBrms	107-184 dBrms	1-10%		No	Hooded seals (*Cystophora cristata*)	Marine mammal	4	34% increase in heart rate compared to baseline	Metabolic	Captive	Kvadsheim et al. 2010
Sonar	downsweep at 3 kHz, total range 2.5-20 kHz		203 dBrms		214	No	Bottlenose dolphin (*Tursiops truncatus*)	Marine mammal	1	TTS from repeated exposure	Hearing	Captive	Mooney et al 2009
Sonar	4.1 kHz		181		199	No	Harbor seal (*Phoca vitulina*)	Marine mammal	1	PTS at 5.8 kHz of at least 8 dB; TTS at 4.1 kHz with recovery after 48 h	Hearing	Captive	Reichmuth et al. 2019
Sonar	3 kHz tone		130-201			No	Bottlenose dolphin (*Tursiops truncatus*)	Marine mammal	1	No significant changes in neural-immune measures	None	Captive	Romano et al. 2004

Journal of Experimental Biology • Supplementary information

Sonar	tones at 0.4, 3, 10, 20, 75 kHz		192-201			No	Beluga whale (*Delphinapterus leucas*) & bottlenose dolphin (*Tursiops truncatus*)	Marine mammal	5 Tt 2 Dl	TTS at 3-20 kHz, variable responses at 75 kHz, no TTS at 0.4 kHz	Hearing	Captive	Schlundt et al. 2000
Other	Drilling from real recording		107-175 dBpp			No	Great scallop (Pecten maximus)	Invertebrate (bivalvia)	Larvae	Increased mortality of pedveliger larvae	Survival	Captive	Gigot et al. 2023
Other	recirculating aquaculture system recording	128 dB				No	Whiteleg shrimp (Litopenaeus vanname)	Invertebrate (crustacea)	300	No effect on metabolic stress, growth or survival measures	None	Captive	Slater et al. 2020
Other	Recording of drilling		Max 167	100% for 12 h		No	Cuttlefish (Sepia officinalis)	Invertebrate (cephalopoda)	20 adults, 30 larvae	Hair cell and lateral line damage; reduction larval hatching success and increase in larval mortality	Hearing, Reproduction	Captive	Solé et al. 2022
Other	white noise		158			No	Goldfish (Carassius auratus) & catfish (Pimelodus pictus)	Fish	12Ca, 19 Pp	TTS with recovery within 3-14 days	Hearing	Captive	Amoser & Ladich 2003
Other	white noise (100-1500)		130, 140 & 150			Yes	Zebrafish (Danio rerio)	Fish	44	TTS with recovery within 7-14 days depending on noise level; hair cell loss	Hearing	Captive	Breitzler et al. 2020
Other	broadband signal from underwater explosion		226-250 dBpeak	single detonatio n	194-212	No	Pacific sardines (Sardinops sagax)	Fish	209	Fat hematoma, and ruptures of reproductive blood, swim bladder and kidney	Injury	Field	Dahl et al. 2022
Other	broadband signal from underwater explosions		Pmax relative to hydrostatic pressure reported	single explosion		No	Pinfish (Lagodon rhomboides), spot (Leiostomus xanthurus)	Fish	190 Lr, 175 Lx	Increased mortality and injury; more pronounced for spot	Death, injury	Field	Govoni et al. 2008
Other	broadband signal from underwater explosion		226 dBpeak	single explosion	196	No	Pacific mackerel (Scomber japonicus)	Fish	253	Increased mortality, damage to the swim bladder (rupture, blood pooling and bruising), kidney rupture and blood pooling in abdominal cavity	Death, injury	Field	Jenkins et al. 2022
Other	white noise (0-1500)		130 & 150			Yes	Zebrafish (Danio rerio)	Fish	3-5 groups of 50 larvae	Increased mortality, cardiac rate, yolk sac consumption and cortisol levels	Death, metabolic	Captive	Lara & Vasconcelo s 2021
Other	10 Hz with harmonics		142-169 dBrms			No	Atlantic salmon (Salmo salar)	Fish	106	Increased plasma cortisol, decreased neuronal activity in hypothalamic tubercula nuclei	Stress, neurological	Captive	Oppedal et al. 2025
Other	white noise (0.3–2.0 kHz)		142			No	Bluegill sunfish (Lepomis macrochirus)	Fish	48	No statistically significant change in auditory threshold	None	Captive	Scholik & Yan 2002b

Journal of Experimental Biology • Supplementary information

Other	100-1000		133 dBrms			No	Atlantic cod (Gadus morhua)	Fish		Reduction in egg production and fertilization rate	Reproduction	Captive	Sierra-Flores et al. 2015
Other	Sea pen recordings	127 dB				No	Atlantic salmon (Salmo salar)	Fish	120 parr	No effect on growth, metabolic parameters, or survival	None	Captive	Slater et al. 2020
Other	white noise 100-10000		170 dBrms			No	Goldfish (Carassius auratus)	Fish		Significant TTS across tested frequencies	Hearing	Captive	Smith et al. 2006
Other	broadband signal from underwater explosion		217 – 252 dBpeak	single detonatio n	193 - 215	No	Pacific mackerel (Scomber japonicus)	Fish	363	Decreased hair cell bundle density	Hearing	Field	Smith et al. 2022
Other	broadband ambient + 25, 29 and 58 Hz tones		115, 130, 150 dBrms	100%		No	Rainbow trout (Oncorhynchus mykiss)	Fish	variable; ~10s	No hearing changes, growth, stress or survival effects	None	Captive	Wysocki et al. 2007
Other	'White noise' with highest energy at 380 Hz		155			No	Hybrid sturgeon (Acipenser baerii × Acipenser schrencki)	Fish	12	Decreased reactive oxygen species in liver tissues, change in regulation of 588 genes, varied effects on protein activity related to liver	Metabolic	Captive	Zhang et al. 2022
Other	AHD pings: 14 kHz with harmonics		98-132 dBrms	Pinging at random intervals		No	Habror porpoise (Phocoena phocoena)	Marine mammal	2 juveniles	Tachycardia in one and bradycardia in the other; respiration rate unchanged.	Metabolic	Field	Elmegaard et al. 2023
Other	broadband signal from arc-gap transducer		~140-182 dBrms		~125-163	No	California sea lion (Zalophus californianus)	Marine mammal	2 males	No physical injury or TTS >6dB observed	None	Captive	Finneran et al. 2003
Other	1/6 octave noise centered at 40 kHz		126-153	100%	162-189	No	Harbor seal (Phoca vitulina)	Marine mammal	2 females	TTS at 1-4 or 12-16 min post exposure, recovery within 2 days	Hearing	Captive	Kastelein et al. 2020

Journal of Experimental Biology • Supplementary information
